# Food Waste and Power Relations in the Agri-Food Chain. The Fruit Sector in Lleida (Catalonia, Spain)

**DOI:** 10.1007/s10806-023-09908-8

**Published:** 2023-05-26

**Authors:** Jordi Gascón, Cristina Larrea-Killinger, Carlota Solà

**Affiliations:** 1 Department of Social Anthropology & Observatori de l’Alimentació ODELA, University of Barcelona, Barcelona, Spain; 2 School of Anthropology and Museum Ethnography, University of Oxford, Oxford, Great Britain

**Keywords:** Food waste, Agriculture, Food chain, Farmers, Lleida (Catalonia, Spain)

## Abstract

Studies of food waste claim that its main causes are technological and logistical deficiencies in the first stages of the agri-food chain. The present article discusses this statement using a specific case as a starting point: the production of fruit in Lleida (Catalonia, Spain). Since the 1980s, fruit production in this region has undergone a process of innovation and development. However, the agents who participate in the sector claim that the wasted volume of edible foodstuffs is greater than in previous times. This article argues that studies of food waste do not consider the power relations existing within the agri-food chain. When these relations are asymmetrical, technological innovation and logistics optimisation do not improve the efficiency of the system; rather, they help the hegemonic players to monopolise the commercial margin and transfer some of their running costs to the weaker agents. The ethnographic data for the study were obtained between 2017 and 2019 using qualitative research techniques.

## Introduction

Most of the academic and institutional literature claims that technological and logistical deficiencies are the main cause of food waste (FW) in the first stage of the agri-food chain, i.e., during production (Parfitt et al., 2010). More specifically, the literature stresses the lack of strategic coordination between different agents in the chain (Muriana, 2017; Arias & Moors, 2018), insufficient investment in technology that can reduce the waste produced during manipulation and processing (Raak et al., 2017; Corradini, 2018), and the use of obsolete production modes on fruit farms (Artiuch & Kornstein, 2012; Lanfranchi et al., 2014). This line of reasoning is defended in the FAO’s report Global Food Losses and Food Waste (Gustavsson et al., 2011), although this document also states that, in high-income countries at least, the principal causes of food waste are to be found in consumer behaviour and the lack of coordination among retailers and that in fact advanced agriculture in these countries results in relatively limited food waste during production.

However, local and regional studies of FW in agricultural production in high-income countries have shown that an enormous volume of food is also lost in this stage (Montagut & Gascón, 2014). Indeed, in recent years, the agriculture sector in these countries has begun to be considered a ‘hotspot of wastage’ (Bos-Brouwers et al., 2014). The EU Fusions Programme^1^ estimated that within the European Union, 30% of waste accumulates during the production and processing stages (Stenmarck, Jensen & Quested, 2016). These findings have not led to a rejection of the FAO argument. In fact, the EU Fusions Programme believes that the accumulation of food waste in the production stage shows that the agricultural sector in these countries is still affected by technological inefficiency (Priefer et al., 2016).

Although we do not deny the role that this technological factor plays in the FW phenomenon, we argue that the academic literature has left out another factor that, in our view, is especially significant: the power relations between the different agents in the agri-food chain. These power relations are asymmetrical and have become even more marked in recent decades as a result of the increasingly disproportionate influence accumulated by large retail food chains and exporting companies (Isakson, 2014). In spite of this, with a few exceptions (Stuart, 2009; Montagut & Gascón, 2014; Gascón, 2018; Bowman, 2020; Gascón, Solà and Larrea, 2022; Hoehn et al., 2023), studies focusing on FW have not addressed the impact of power relations within the agri-food chain on FW accumulation.

Political ecology takes into account the characteristics of the hegemonic agri-food model, and an approach from this perspective offers an opposing hypothesis: what if the origin of FW is to be found in this model and in the unequal power relations that characterise it? (Gascón, 2018) Political ecology introduces power relations into production processes in two ways. First, it examines the consequences of these relations: political ecology warns that production processes may entail “externalities”, that is, social costs that are not considered or measured by the market. Externalities do not affect all social sectors in the same way since their impact is strongest on the most vulnerable sectors (Martínez Alier, 1995; Escobar, 2016).

Second, political ecology focuses on the causes of power relations. Not all agents involved in the production process take on the same responsibility for their unintended and uncalculated impacts. The market conceals the responsibility of different agents because it ignores these externalities (Martínez-Alier, 2003; Leff 2015). This is the point that interests us in this paper. Analysing FW from the political ecology perspective compels us to ask which agents bear the most responsibility for it. Specifically, **our hypothesis** is that when the relations within the agri-food chain are asymmetrical, technological innovation and logistical optimisation do not necessarily improve system efficiency, understood as the best use and exploitation of the resources available. As we will show, this is because hegemonic agents, i.e., the large retail chains and exporting companies (LRC&EC), have the opportunity to monopolise the commercial margin and transfer their running costs to the weaker agents in the system. Farmers, for example – weak agents within the conventional agri-food system – may be forced to modernise their production methods by introducing strategies that generate FW. In other words, production is not planned to maximise the use of farm resources but rather to fulfil the interests of large retail chains and exporting companies.

Our research focuses on the central area of the province of Lleida (Catalonia, Spain), the region of Spain with the highest production of apples and pears and very high levels of peach production. In 2018, fruit production occupied an area of 35,172 hectares (DARP, 2019). The fruit sector in Lleida consists of the farmers, the collection centres, and the LRC&EC. As in most parts of the agri-food system (McMichael, 2009), the last group has become the key stakeholder. Since the 1980s, fruit production in Lleida has undergone an intense process of technological and logistical innovation. However, both farmers and managers of the collection centres report an increase in the percentage of healthy produce that is rejected and does not enter the agri-food circuit. The present article analyses whether this phenomenon is due to the ability of large retail chains to impose prices and conditions on the producer. If so, how does this process take place? We focus on conventional and integrated agriculture, which makes the largest contribution to the territory’s fruit production (97% in 2019, Observatori de l’agricultura i l’alimentació ecològiques, 2020).

## Methodology

Fieldwork was carried out with farmers, seasonal workers, and the technical staff of collection centres for the five fruit sectors in the province of Lleida. We used an ethnographic research method (Bernard, 2017) that explores social behaviour through qualitative research techniques: participant observation (joining the workers picking fruit during harvest), semistructured interviews (more than 20), informal conversations, and focus groups. The in-depth interviews and focus groups aimed to (re)construct the experiences of the participants in detail. All data collected were collated in the field diary. The analysis was longitudinal since the authors conducted several research stays over the course of the study period. The first stay, during the summer of 2017, lasted three months and was followed by several shorter research stays up until 2019.

Although the fieldwork continued until 2021, data recorded after the onset of the COVID-19 pandemic were not included due to its likely impact on FW during the period.

## Lleida and the Fruit Sector

Spain’s entry into the European Union in 1986 brought in a series of new agricultural guidelines, which in turn called for the restructuring of the agricultural sector (Majoral, 2006; Clar, 2017). Farming in Lleida adapted to the new situation by applying structural changes, such as the irrigation of almost all fields (Observatori de la Fruita, 2019) and the introduction of a network of refrigerators (Ruiz et al., 2003). However, in relation to the topic of this article, the most notable change was the process of business concentration. Greater horizontal integration favoured the concentration of land, the creation of collection centres (both cooperatives and private wholesalers) and larger distribution structures. At the end of the 2010s, the concentration of property ownership in Lleida was 7 CV/H (cadastral value/number of holders), twice the Spanish average, and the distribution centres co-opted 80% of the fruit produced in the region (Mallada & Colom, 2010). This strategy of horizontal integration aimed to increase the competitiveness of the fruit in the European market (Pascual et al., 2006).

This transformation process was led by supermarkets, exporting companies and associations of shops with a single sales point. Furthermore, changes in consumption patterns, which favoured supermarkets and greengrocer chains over small food stores, played an important role in this process. The large retail chains accumulated a very high percentage of the total number of fruit sales, creating a bottleneck for producers: most farmers could only reach the market through these large retail chains. The relationship of dependency that this caused meant that the LRC&EC could dictate prices and impose conditions on the farmers regarding the production process (Farré & Sala, 2014).

The hegemony of the LRC&EC in the agri-food chain also freed them from oversight of collecting and homogenising farmers’ production. The large retail chains externalised these operations to the collection centres, which collect and manage their stocks of produce. In the early 2010s, these centres co-opted 80% of the fruit produced in Lleida (Mallada & Colom, 2010). Formally, the role of these agents is to favour the organisation of production and its commercialisation. However, they have an additional role that is equally important: namely, they communicate the demands of the LRC&EC to the farmers.

**Fig. 1:**
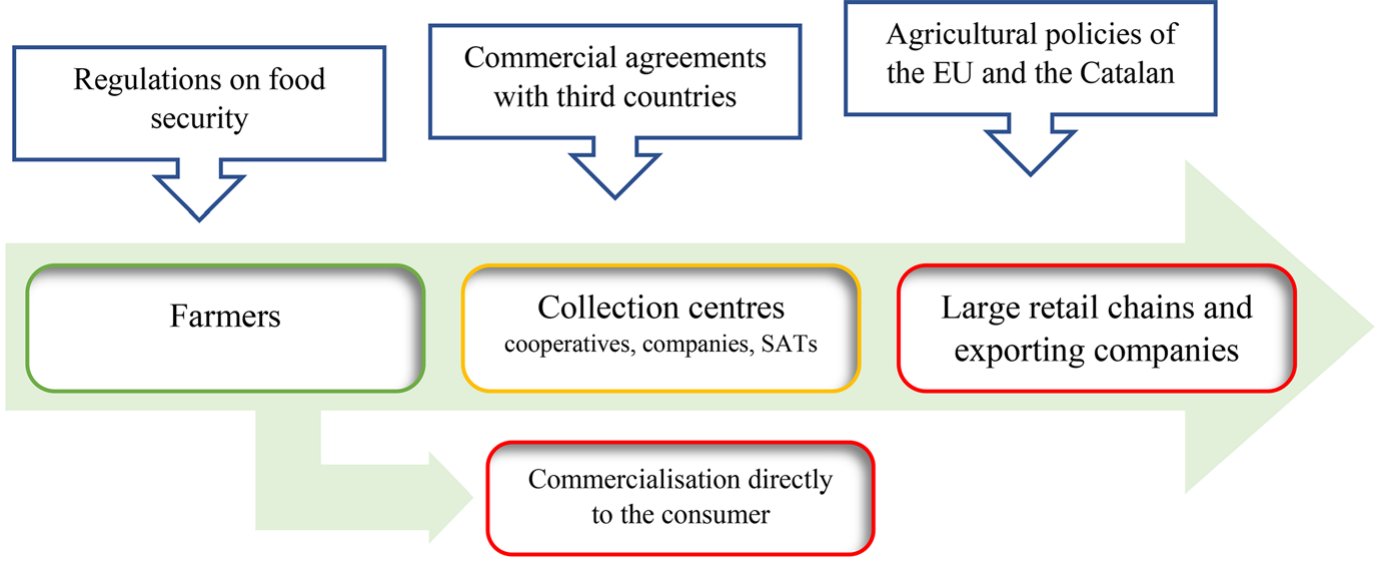
The Lleida fruit sector

Our thesis is that the power inequalities within the sector of farmers, collection centres and large retail chains have consequences for the accumulation of FW in Lleida. Public institutions are also key stakeholders that should be included in the analysis, given the importance of their policies regarding agriculture, food safety and the opening of markets (Fig. 1).

The inequality of these economic relations underlies several processes that generate FW. These processes can be grouped into two categories. The first category comprises the conditions imposed by LRC&EC and, to a lesser extent, public institutions that oblige farmers to adopt processes that remove edible produce from the agri-food circuit. The second comprises the strategies that farmers are forced to deploy to reach a market over which they have very little influence as a result of the inequality of the power structure.

## Conditions Imposed by Large Retail Chains and Exporting Companies

### Quality and Appearance

As far as fruit quality is concerned, the conventional commercialisation system prioritises its appearance (calibre, texture, colour and shape) over its nutritional value or organoleptic properties. Visual irregularities are not accepted, even if they do not affect nutritional quality. These requisites, which aim to standardise the production entering the agri-food circuit (Konefal et al., 2005), are imposed by the LRC&EC. However, it would be wrong to regard them simply as a whim; they are the result of the logistical structure of the large retail chains and exporters, which need the produce to ripen homogeneously after being picked to properly control their storehouses. The produce is often collected in boxes containing trays with indentations (especially in the case of the most fragile fruit), and therefore, the pieces need to be of a similar size to fit in the indentations. The requirements that the LRC&EC place on farmers are also the result of the sales system. In supermarkets and greengrocer chains, the task of choosing products and placing them in bags has been externalised to consumers. If the fruit has a very heterogeneous appearance, consumers choose the ones that are most attractive, and the other pieces accumulate until they rot. Consequently, the logistics system of exporters and large retail chains is possible insofar as its hegemonic position allows it to transfer part of its running costs to the production and collection stages.

In addition, distributors’ quality requirements vary. Producers and collection centres highlight two elements that can explain these variations. One of them is the average appearance of the produce in the market. This may depend on supply and demand: if the harvest has not been abundant, more irregular products with smaller calibres will be accepted. Similarly, at the beginning of the harvest season, when there are still only a few products on the market, the quality control will not be as strict. The second aspect is the fact that collection centres do not establish the same quality requirements: some of them work with second class markets, which are less demanding and accept a greater degree of irregularity in the produce but are consequently cheaper.

As a result, the produce goes through three screening stages before reaching the storehouses of large retail chains and exporters. The first one takes place on the farm. At the beginning of the harvest, the harvesters receive instructions about the visual quality of the fruit, which will depend on the requirements of the collection centre to which the production will be taken, as well as each farmer’s own strategy. As we will see, this strategy depends on factors such as the availability and cost of labour and the size of the farm. Therefore, harvesters not only leave fruit that is rotten or has been attacked by pests but also pieces that do not meet the specified visual and size requirements. Furthermore, pieces that are too ripe or have slight scratches are also rejected since they would not withstand the transportation and storage processes. All our informants (managers of collection centres and farmers) agreed that in the local markets that prevailed until the 1950s, this produce could be sold without any problem.

The second screening takes place in the collection centre. Cooperatives and companies have the power to withdraw fruit that does not meet quality requirements. Their role as inspectors for exporters and large retail chains obliges them to be strict with producers. Nevertheless, as we noted above, the requirements are not always the same and may depend on the particular markets. Finally, the LRC&EC carry out the third screening on the produce offered by the collection centres. As the centres are keen to continue supplying the LRC&EC, the quality of the produce that reaches the storehouses of the large retail chains usually meets the latter’s requirements. The collection centres prefer to put pressure on the farmers, who depend on them, rather than to negotiate with the LRC&EC, on whom they depend.

The fruit that is withdrawn does not necessarily leave the agri-food system. There is in fact a market for this produce, namely, the juice and purée industry. Except for pieces that are rotting, in theory, all the produce rejected in the procedure described above can go to these industries. In practice, however, this is not the case. In the late 2000s, Mallada and Colom (2010) calculated that only 6% of the produce from Lleida went to juices and purées. As we will see later, our calculations show that the percentage of perfectly edible fruit that does not enter the fresh fruit system is actually much higher.

Two factors can explain why most of the withdrawn fruit is not used in the juice and purée industry. The first is that not all fruit varieties are accepted. Peaches are a case in point. In the late 2010s, red peaches were the most sought-after fruit in the fresh circuit. However, the juice industry would not accept them; the colour of their juice is red, and the large retail chains have established that peach juice must be yellow. The second factor is the price.

### Price

The hegemony of the LRC&EC in the fruit sector in Lleida means that they can impose their prices on farmers. Other factors affecting prices are the increase in supply from the European Union due to the incentives of the Common Agricultural Policy (CAP), the expansion into eastern European countries with strong agricultural sectors such as Poland, and the signing of commercial treaties with countries in the global South with low production costs, which allows them to introduce fruit into the European markets at competitive prices. In this context, large retail chains and exporters can increase their profit margins at the expense of local producers. Therefore, the tendency for sales prices at the origin is to decrease. In the case of apples and pears produced in Lleida, in 2017, the difference between the production price and the end-consumer price was higher than 400%. For peaches and nectarines, the difference reached 1,100%. (see Table 1).

**Table 1:** Production price and end-consumer price of the main fresh fruits in Lleida (comparison between 2008 and 2017)

Produce	Central month of the season	2017		2008	
		**Price at origin** (euros/kg)	**Price at destination** (euros/kg)	**Price at origin** (euros/kg)	**Price at destination** (euros/kg)
Apple	October	0.45	1.95	0.33	1.80
Pear	September	0.44	1.70	0.80	2.26
Peach	August	0.15	1.75	0.55	2.24
Nectarine	July	0.15	1.73	0.65	2.16

Source: COAG (2020)

In contrast, production costs continue to increase. In these conditions, the need to balance income and expenditure is the second difficulty farmers face, in addition to meeting the homogeneity requirements during the production process. In fact, there have been seasons in which the end-consumer price established by the large retail chains is lower than the production cost (Iglesias & Casals, 2011).

This tension is reflected in the FW. More specifically, this is the reason why most of the production withdrawn from the fresh fruit circuit does not go to the juice and purée industries. During our fieldwork, the purchase price of fresh peaches bought from farmers was approximately 0.18 euros/kg, depending on the month of the year and the quality of the fruit, but the purchase price of the fruit for juice or purée was only 0.03 euros/kg. The collection centres blamed this low price on the abundance of peaches (higher than demand) in the international market.

Farmers pointed to another cause, namely, the structures and policies of the collection centres. With the selection made by the collection centres, approximately 20% of the fruit brought in by farmers is withdrawn from the fresh fruit circuit. For the collection centres, it is more profitable to sell this fruit to the industries at a lower price rather than to manage it in other ways (for example, by burning or as compost). However, for most farmers, it is more profitable to leave this produce on the ground than to collect it since the established price does not cover the labour costs; the fruit is mulched using the shredding machine and used as fertiliser for the next season. However, this practice is not widespread, and each farmer has a different strategy. For instance, for small fruit farms run exclusively by families who do not hire any temporary workers, it is worth their while to collect all the fruit considered adequate for the juice and purée industries.

The relationship between labour price and sale price is also reflected in the care with which the produce destined for the fresh market is collected. The instructions farmers give their workers depend on this factor. If the price is very low, farmers do not usually want workers to spend too long inspecting the fruit, which means that a considerable amount of fruit that would have been suitable for the fresh market goes to waste. Again, the fruit farms run exclusively by family members tend to allocate more time to this task, which in turn reduces the total FW.

### Markets

The distance between the place of production and place of consumption affects the accumulation of FW. This is frequently attributed to problems related to transportation and storage processes (Milà et al., 2007; Wakeland et al., 2012). The case of fruit in Lleida demonstrates that the distance between the place of production and the final market also affects the accumulation of FW during the harvesting stage. For instance, fresh fruit destined for distant markets through exporting companies needs to be harvested before it is fully ripe so that it will withstand the transportation process and have an optimal appearance at the sales point. If the fruit has already reached this level of ripeness on the tree, it will be rejected because it will reach the supermarket in poor condition. Similarly, the fruit cannot have any scarring, as this can lead to rotting if it is consumed in the mid-term. This was not a problem when the produce was commercialised in local markets. As noted above, the produce considered fragile and of good quality must be transported in boxes with trays with individual indentations, meaning that the pieces must have a uniform size.

Fortunately, different markets have different demands. For instance, farmers report that consumers in the United Kingdom pay more attention to the colour of the fruit, those in Spain ask for large sizes, and thoe in Germany focus on the phytosanitary residues in the fruit rather than its appearance. Therefore, some collection centres working with a broader range of clients can be less restrictive with their producers, although this may entail a lower purchase price. In some cases, they can establish up to three categories of fresh fruit, apart from the category destined for juices and purées. However, the collection centres working with only one client profile have to be more demanding about the requirements imposed on farmers, which increases the amount of produce left to rot in the fields.

Farmers are aware that the new hegemonic markets cause more FW than local traditional markets. In addition, local markets are disappearing due to the hegemony of supermarkets in the commercial circuit, against which they have become marginal. Only a few traditional local markets remain, in which a limited number of farmers are able to participate. In the last decade, there has been a rise in alternative pathways to consumers, such as farmers’ markets, but these are usually supplied by ‘organic farmers’ rather than those practising conventional or integrated farming.

## Positioning Strategies in the Market

The strategies that farmers deploy to reach the market – in a sector where they are the weakest agents – constitute the second group of factors that influence the generation of FW. Farmers establish different production logics depending on different variables: the type of labour force available (family-based or hired), the size of the property, whether the investments have already been paid off, the characteristics of the market for which they work, the fluctuating price of the fruit, and their intuition about the mid- and long-term market situation. Importantly, not all of these variables have the same consequences for FW.

### Production Structure

Not all the conventional farmers in Lleida have the same productive structure, nor do they apply the same strategies. The most influential factor on the accumulation of FW in the fields is the type of labour force available to the farmer. Whether the fruit that might be destined for the juice or purée industry will be collected depends on the type of labour force, as well as whether the harvesters spend enough time properly selecting the produce on the tree. On some occasions, the workers (or at least some of them) are family members, but often there is an increase in hired labour for the harvest.

Two production models emerge from the data collected in the study. The first corresponds to medium-large fruit farms (those with over 40 hectares), which are usually technically advanced and which hire day labourers. The strategy of this model is to obtain the largest amount of fruit suitable for the market at the lowest cost/hour/labourer. In these cases, the relative amount of fruit destined for the juice and purée industries is usually small: the price of the fruit does not compensate for the cost of the labour needed to collect it. The harvest is also carried out as quickly as possible, so the selection of the fruit on the tree may not be done with great care: it is more profitable to lose part of the harvest than to increase the working hours of the labourers. Similarly, the care of the fruit trees (pruning, scions, etc.) before and after harvest may also be substandard and thus affect tree production.

The second type of model corresponds to smaller farms, although the numbers of these farms are decreasing as a result of generational renewal and the forces that encourage the concentration of land. These small fruit farms still rely heavily on the family labour force, take greater care of the trees and select the fruit more meticulously during the harvest, as has also been observed in other geographical settings (Bentley, 1990). For instance, it is common for these fruit farms to carry out more ‘passes’ (the times the harvesting team picks fruit from the same area, collecting only the produce that is ripe enough). Increasing the number of ‘passes’ ensures that a substantial part of the harvest will meet the calibre and maturation requirements demanded by the collection centre; however, it also increases the cost of labour if the labour is contracted.

The involvement of family members presents two advantages in relation to FW. On the one hand, it increases the quality of the harvest: the workers are specialised in the production of fruit, know how the farm works and are keen to obtain the highest possible yield. In contrast, hired workers are usually untrained, which entails the loss of a large part of the harvest. Above all, these workers cannot carry out their tasks efficiently due to time pressure.

The second competitive advantage of family-based fruit farms is that their economic philosophy is not the same as that of modern fruit farms, where the labour force has no attachment to the property and needs to be paid. This means that these small producers can function under conditions in which standard capitalist enterprises would go bankrupt. Family-based agriculture does not need to generate a profit because the value of the resources does not reside in the benefits but rather in ensuring the reproduction of the domestic group. As a result, they make the most of the available labour, even in situations that would cause a negative output in a larger business (Shanin, 1973; Chayanov, ([1966]1986). In the present case, this implication in the task at hand leads to better care of the trees and the harvesting process and, consequently, to a lower accumulation of wasted food. In the fields of Lleida, this concept can be seen in practice. When we asked the manager of a collection centre about the farmers who collected the fruit for the juice industries, he said, ‘maybe the farmers who pick that fruit don’t use hired labour’. It is also common to hear that those who work with family labour take better care of the trees, which allows them to reduce the negative impact of pests.

However, there is one economic strategy that has a strong impact on FW. Antoni and his wife are conventional farmers who manage a small fruit farm (25 hectares). They send part of their produce to the cooperative, but they sell the rest in the local market in Lleida where they have a stall. Combining production and commercialisation has two advantages. First, it is feasible in a fruit farm that is not too large, since the produce that they sell directly to the consumer allows them to obtain the commercial margin that would be absorbed by the large retail chains; the earnings that small farmers make when they sell directly in the market are much higher. Therefore, it is beneficial for them to allocate more time to caring for their farm. Second, having a stall in the market allows them to sell produce that, due to its calibre or shape, would not be accepted by the large retail chains.

### Overproduction

Our respondents also indicated that the competition between farmers is another risk factor for FW. When a fruit variety has a market and a ‘good’ price, many farmers decide to grow it; as a result, the supply of this fruit rises and the price falls. The problem is that farmers plan their production process without knowing the future conditions of increasingly globalised markets. In this situation, their fear of being left out of a competitive market leads them to increase their productivity (i.e., produce per unit of land). If farmer A succeeds in this endeavour, farmer B loses competitiveness and can become marginalised from the market. This spiral is currently accelerating, stimulated by productivist public policies and an obsession with growth.

One consequence of this process is the risk of overproduction (Serrano & Pinilla, 2010). The surplus fruit cannot be introduced into the market because this would cause prices to plummet and thus ruin the farmers. In the past, the produce that was withdrawn was burnt. However, the new policy of the European Union establishes that, as far as possible, the withdrawn fruit should either be distributed for free through care programmes or destined for use as animal feed or compost; see Table 2 (European Parliament, 2017).

**Table 2:** Volumes withdrawn in Spain of the main fruits produced in Lleida according to their destinations, in tonnes (Season 2019)*

Produce	Free distribution	Animal food	Other destinations (compost)
Apple	420	0	0
Pear	567	2	0
Peach	3,137	695	323
Nectarine	3,542	807	472
Saturn peach	4,290	666	83

Source: MAPA-FEGA (2019)

* Overall data for the whole of Spain; no data are available for individual autonomous communities

The official data do not consider the produce destined for compost as FW. This is because the definition of FW used by public European institutions calculates it according to volume: one tonne of produce equals one tonne of supplies for compost. There is a one-to-one correlation in volume only. However, there is no equivalence between the energy cost invested in the production of a piece of fruit and the value that it can return as compost; in contrast, this is a negative correlation. The energy cost of producing an apple is much higher than the energy that this apple can provide as part of agricultural supply. Using food produce that cannot enter the market for compost reduces the metabolic rift, but it does not compensate for it (Cuéllar & Webber, 2010; Gascón, 2018).

Nevertheless, the greatest problem posed by overproduction with regard to FW is the tightening of the quality requirements established by large retail chains and exporting companies. As we have seen, at the beginning of the season, when demand is higher than supply, the quality requirements are less strict. However, this is so only during a very short timeframe and affects a minimal part of the annual production. Due to overproduction, by the end of the year, the quality requirements established by large retail chains are so strict that a large part of the overall produce is excluded from the agri-food circuit. Although it might seem paradoxical, the relationship between price and supply in the market resulting from overproduction leads farmers to demand stricter quality requirements regarding visual quality. Exporting companies and large retail chains establish these requirements to transfer part of their technical and logistical costs to the previous stages of the agri-food circuit. Farmers report that they demand the same requirements to reduce the fruit entering the market and consequently avoid a decrease in price.

## Conclusion

According to Hodgins and Parizeau (2000), the extensive literature on power relations in globalised food systems has not generally addressed the phenomenon of FW, with some exceptions (Pimentel, 1990; Gascón, 2018). Most of the literature analysing FW during the first stages of the agri-food chain attribute it mainly to logistical and technological shortcomings (Soysal et al., 2012; Göbel et al., 2015; Díaz et al., 2018). This line of thought was established and legitimised by the FAO (Gustavsson et al., 2011) and has been accepted either implicitly or explicitly by other multilateral institutions such as the European Union (Östergren et al., 2014). Nevertheless, our analysis of the fruit sector in Lleida shows an inverse relationship: the accumulation of FW grows in parallel with the increasing investment in innovation and development.

We argue that the problem of the aforementioned studies is that they mistake output for efficiency: in the fruit sector in Lleida, technological innovation and logistics optimisation have aimed to increase company profits (output) rather than to make the most of the resources available (efficiency). The fruit sector in Lleida is an asymmetric productive and commercial structure in which the LRC&EC accumulate oligopolistic power. This hegemony allows them to impose conditions on the other agents of the sector, the farmers and collection centres. In this context, they obtain their profits not by improving the efficiency of the agri-food chain but by monopolising most of the commercial margin and transferring part of their own running costs to the weaker agents in the chain. In addition, these conditions generate FW in the field. As we have shown throughout the text, the low price of the fruit forces farmers to reduce the labour force needed to obtain an optimal use of the land. Furthermore, the quality requirements of the produce make it impossible for farmers to introduce perfectly edible food into the commercial circuit. Finally, the inability to influence the market forces farmers to deploy inefficient strategies, such as overproduction, to be able to enter the market. Thus, in the case of the fruit sector in Lleida, the strategies used by each agent are not irrational, based on poor logistical planning, or due to technological deficiencies but result from the unequal power relations that oblige farmers to establish an inefficient agri-food model that in turn accumulates FW.

Of course, farmers, who are aware of their subordinate position, respond to the sector’s structure in different ways; they have room to manoeuvre. Adopting a particular strategy positively or negatively affects the accumulation of FW. For instance, some farmers try to move away from the hegemonic market. Although our analysis has focused on conventional and integrated farming, it is worth noting that generally the farmers who try to move away from the hegemonic market also try to become organic farmers (at present, in the organic market, the relations between the agents of the agri-food chain are more egalitarian). Others who maintain conventional or integrated farming methods aspire to commercialise their produce directly with the end consumer through local and farmers’ markets. These strategies are included in the process known as repeasantisation: the search for greater autonomy to avoid a market that relegates farmers to a position of extreme vulnerability (Ploeg, 2008; Akram-Lodhi & Kay, 2010). Those who are successful reduce their FW since they are not bound by the quality standards required by the large retail chains and can sell all their produce, whatever its size or colour. Furthermore, since farmers obtain all the commercial margins of the fruit, they are particularly keen to minimise waste. However, few farmers are able to enter this market; it is small and unable to absorb a large percentage of the total amount of fruit produced in Lleida. In addition, the medium and large size of most fruit farms compels farmers to concentrate on their productive tasks, resulting in production volumes that are too large to be commercialised by retailers.

The size of the farms and the type of labour available determine the farmers’ strategies. In turn, these strategies have different effects on the accumulation of FW. Although fruit production in Lleida has become almost a monoculture, not all fruit farms have the same characteristics. The main objective of small and medium-sized farms, where the labour force is predominantly family-based, is not to generate a large profit but to ensure the reproduction of the domestic group (Shanin, 1973; Giner & Sevilla, 1980; Chayanov, ([1966]1986). This provides them with a comparative advantage over the larger farms that depend on hired labour. Small, family-oriented fruit farms can be viable with a yield that would not allow a capitalistic enterprise to survive. As explained above, labour in the capitalistic enterprise is a running cost; however, in family-based agriculture, it is a benefit. This has consequences for the FW in the region under study: family-based agriculture takes greater care of the farm, so its resources and production are used more efficiently.

Studies in the literature quite often claim that in Western countries, the production and consumer processes are the factors most responsible for FW (Gustavsson et al., 2011; Priefer et al., 2016); however, our research on the fruit sector in Lleida leads us to a different conclusion. Although a substantial part of the food that does not reach our tables lies rotting in the fields, the responsibility for this lies not so much with the farmers as with the conditions imposed by a market that they do not control and with the interests of the agents that monopolise power in the agri-food system.

## Data Availability

The datasets from this study are available from the corresponding author on reasonable request.

## Footnotes

1 The EU Fusions Programme is a European Union programme that promotes research into FW in order to find ways to prevent it.

## Code Availability

Not aplicable.
